# Prognostic value of myocardial T2 mapping post reperfused acute myocardial infarction

**DOI:** 10.1186/1532-429X-17-S1-P150

**Published:** 2015-02-03

**Authors:** Mohammad I Zia, Idan Roifman, Nilesh R Ghugre, Abel J Ignatius, Bradley H Strauss, Alexander Dick, Graham A Wright, Kim A Connelly

**Affiliations:** Cardiology, Sunnybrook Health Sciences Centre, Toronto, ON Canada

## Background

Myocardial edema is known to be a common phenomenon post acute myocardial infarction. The aim of this study was to define the prognostic significance of myocardial edema using cardiac magnetic resonance (CMR) and T2 mapping techniques in patients with reperfused ST-segment elevation myocardial infarction (STEMI).

## Methods

Fifty-four patients were enrolled post primary percutaneous coronary intervention (PCI) and underwent CMR on a 1.5T scanner at 48 hours. Myocardial edema was quantified using a T2 mapping technique, while infarct size was assessed via a contrast-enhanced T1-weighted inversion recovery gradient-echo sequence. Information regarding four clinical outcomes: a) mortality, b) repeat myocardial infarction, c) heart failure hospitalization and d) repeat revascularization was collected at 12 months post index primary PCI. Our primary clinical endpoint was a composite of these 4 outcome measures of major adverse cardiovascular events (MACE). The Cox proportional hazards regression model was used to calculate the relative risk of MACE for increased T2 values, adjusted for baseline characteristics related to increased edema (diabetes status, symptom to balloon time, infarct size).

## Results

The mean age was 59.6 ± 8.2 years, 88% were males, 35% were diabetics, 44% were hypertensive. The mean symptom to balloon time was 395 ± 230 minutes and mean door to balloon time was 81.5 ± 39 minutes. The mean T2 value was higher in the infarct segment compared to remote segment (55.1 ± 7.6 ms vs 40.2 ± 2.6 ms, p<0.001). Patients with MACE (n=12) had higher infarct segment T2 values compared to patients that did not (64.4 ± 7.3 ms vs 51.1 ± 5.8 ms, p<0.001). After adjustment for variables associated with increased edema, a T2 value of ≥ 62 ms was associated with a hazard ratio of 1.04 (95% CI:1.01-1.07, p=0.009) for MACE at 12 months (Table 1).

**Table 1:** Multivariate Analysis for the Determinants of MACE at 12 Months

VARIABLE	Hazard Ratio (95% CI)	p Value
Diabetes	1.01 (0.96-1.05)	0.24
Symptom to Balloon Time	1.00 (0.98-1.03)	0.19
Infarct Size	1.03 (0.98-1.06)	0.26
Infarct Segment T2 (>62ms)	1.04 (1.01-1.07)	0.009

## Conclusions

In reperfused STEMI patients, higher T2 values in the infarct segment are associated with worse prognosis. This suggests the need to develop therapies aimed at reducing myocardial edema post acute myocardial infarction.

## Funding

Dr. Connelly is supported by a Heart and Stroke Foundation of Canada Phase 1 Clinician Scientist Award. Dr. Dick is supported by the Heart and Stroke Foundation of Canada. Dr. Strauss holds the Reichmann Chair in Cardiovascular Research. Dr. Wright receives research funding from GE Healthcare. This work was supported in part by a Canadian Institutes of Health Research (CIHR) operating grant, the Ontario Research Fund, and GE Healthcare.

